# Neurocognitive testing in a murine model of mucopolysaccharidosis type IIIA

**DOI:** 10.1016/j.ymgmr.2023.100985

**Published:** 2023-06-10

**Authors:** Kleopatra Pericleous, Chantelle McIntyre, Maria Fuller

**Affiliations:** a Genetics and Molecular Pathology, SA Pathology at Women's and Children's Hospital, 72 King William Road, North Adelaide 5006, Australia; b School of Biological Sciences, University of Adelaide, Adelaide 5000, Australia; c Adelaide Medical School, University of Adelaide, Adelaide 5000, Australia

**Keywords:** Mouse model, Lysosomal storage disorder, Sanfilippo A syndrome, Neurobehaviour, Mucopolysaccharidosis

## Abstract

Mucopolysaccharidosis type IIIA (MPS IIIA) is an inherited metabolic disorder caused by a lysosomal enzyme deficiency resulting in heparan sulphate (HS) accumulation and manifests with a progressive neurodegenerative phenotype. A naturally occurring MPS IIIA mouse model is invaluable for preclinical evaluation of potential treatments but the ability to effectively assess neurological function has proved challenging. Here, the aim was to evaluate a set of behaviour tests for their reliability in assessing disease progression in the MPS IIIA mouse model. Compared to wild-type (WT) mice, MPS IIIA mice displayed memory and learning deficits in the water crossmaze from mid-stage disease and locomotor impairment in the hind-limb gait assessment at late-stage disease, supporting previous findings. Declined wellbeing was also observed in the MPS IIIA mice via burrowing and nest building evaluation at late-stage disease compared to WT mice, mirroring the progressive nature of neurological disease. Excessive HS accumulation observed in the MPS IIIA mouse brain from 1 month of age did not appear to manifest as abnormal behaviours until at least 6 months of age suggesting there may be a threshold of HS accumulation before measurable neurocognitive decline. Results obtained from the open field and three-chamber sociability test are inconsistent with previous studies and do not reflect MPS IIIA patient disease progression, suggesting these assessments are not reliable. In conclusion, water cross-maze, hind-limb gait, nest building and burrowing, are promising assessments in the MPS IIIA mouse model, which produce consistent results that mimic the human disease.

## Highlights

• Water-cross maze and hind-limb gait are reliable behaviour tests.
• Sociability and open-field activity measures are variable and not robust.
• Patient quality-of-life can be modelled via well-being assessments in mice.
• Burrowing and nest building show decreased well-being in MPS IIIA mice.
• Heparan sulphate storage appears before behavioural deficits can be measured.

## 1 Introduction

Mucopolysaccharidosis IIIA (MPS IIIA),^1^ also known as Sanfilippo A syndrome, is an inherited autosomal recessive lysosomal storage disorder arising due to a defect in the hydrolytic enzyme, *N*-sulphoglucosamine sulphohydrolase (SGSH)^2^ (EC 3.10.1.1). As SGSH is required for the degradation of the glycosaminoglycan, heparan sulphate (HS),^3^ MPS IIIA is biochemically characterised by lysosomal accumulation of incompletely degraded HS [1]. Primarily a neurodegenerative disorder, with relatively minimal somatic disease, the neuropathology is progressive, signalised by central nervous system dysfunction, significant behavioural abnormalities and neurocognitive decline. Infants with MPS IIIA are typically asymptomatic at birth [1,2] and clinical manifestations are proposed to progress in three stages, eventually advancing to a vegetative state and premature death in the second decade of life [3].

To date, there are no cures or clinically approved therapies for MPS IIIA, with current treatment limited to palliative care and managing behaviour with conventional therapies and medications [4]. Several clinical trials are underway, aimed at addressing the root-cause of the disease by replacing the defective enzyme (NCT03612869, NCT02716246, NCT04201405) and functional improvement is typically ascertained from cognitive and developmental assessments such as the Bayley Scales of Infant and Toddler Development, the Kaufman Assessment Battery for Children, and Vineland™ Adaptive Behaviour Scales [5]. However, demonstration of neurocognitive benefit in MPS IIIA patients is difficult to assess due to patient disruptive/non-cooperative behaviour, dementia, low cognitive functioning and sensory/physical disabilities [6]. Additionally, limited natural history studies have resulted in incomplete knowledge on the natural progression and variability of the disease to create a benchmark for neurocognition.

Prior to clinical trials, preclinical studies are typically performed in a mouse model (B6.Cg-Sgsh^mps3a^) of MPS IIIA, and functional outcome is assessed using behavioural assessments [[7], [8], [9]]. These tests are designed to determine whether therapy improves the neurological phenotype in mice and then interpolate these findings in the context of the human condition. Demonstrating functional benefit in mice paves the way for human trials and helps set realistic expectations for therapeutic goals. Additionally, as therapies must remove the accumulated HS to achieve functional improvement, the relationship between brain biochemistry and neurocognitive function is important. HS accumulation in MPS IIIA mice has been shown to increase in the different regions of the brain as the disease progresses [10]. Behaviour studies in mice can be variable due to differences in environmental factors and equipment, animal handling and investigator interpretations, mouse housing, upbringing and prior (testing) experience [11]. For example, hypoactivity has been reported at 3–8 months of age in MPS IIIA mice [8,12,13] and hyperactivity [14,15] or “normal” activity has been found at 2–9 months of age [9,16,17].

In this study, the aim was to explore which behaviour tests produce clear and consistent results, reflect HS storage in the brain and importantly may be considered surrogate measures of neurocognitive performance in the MPS IIIA mouse model. This was achieved using a battery of behaviour tests assessing MPS IIIA patient behaviours of cognitive skill regression and dementia [18] via memory and learning, hyper−/hypoactivity and impaired locomotion [3] as well as social deficits [19]. Wellbeing in the MPS IIIA mice was also assessed through innate behaviours of nest building and burrowing in an attempt to provide a surrogate measure of life-quality in MPS IIIA patients [20,21]. The behaviour tests were conducted at ages representing early (3 months), mid (6 months) and late stages (8–10 months) of disease resembling the three-stage human progression and were performed within a single facility to remove environmental variability.

## 2 Materials and methods

### 2.1 Animal husbandry

The naturally occurring MPS IIIA mouse model B6.Cg-Sgsh^mps3a^/PstJ (C57BL/6 background) (RRID:IMSR_JAX:003780) was purchased from the Jackson Laboratory (Bar Harbour, ME, USA) and a colony established at the Women's and Children's Hospital Animal Care Facility. This study was approved by the Institutional Animal Ethics Committee (AE1114) in compliance with the Australian Code for the care and use of animals for scientific purpose 8th Edition (2013). MPS IIIA mice were bred from homozygous pairs. Mice were either individually or gender-matched group housed (2–5 per cage) according to genotype and age. All mice were monitored for aggression (particularly group housed males), rectal prolapse and bladder distension and weighed weekly. Mice were housed in a large PC1 holding room in open-top conventional cages (enriched with paper pellet bedding, housing and shredded paper) on a 14/10 h light/dark cycle in a temperature-controlled facility (22 °C) and allowed ad libitum food (regular rodent chow) and water. Mice were separated into three mixed-gender groups for age-associated behaviour testing at 3, 6 and 8–10 months of age.

### 2.2 Water cross-maze

Memory and learning were assessed using the water cross-maze test as detailed previously [22]. Four visual cues were placed around a circular pool containing a platform submerged in clouded milk-powder water. Within the circular pool, the mice were confined to a 4-armed-cross swimming area; one of the arms containing the platform. For five consecutive days, with six trials per day, the time taken to locate the platform (latency), number of entries/re-entries into each arm from the release point without finding the platform (incorrect entries) and number of trials per day a mouse would swim directly to the platform from release point (correct entries) were recorded for each mouse.

### 2.3 Open field

Motor activity was assessed using an open field arena (40 cm × 40 cm) [23]. An automated activity monitoring system consisting of infrared sensors that detect horizontal and vertical line crosses in the arena (Harvard Apparatus, Holliston, MA, USA) generated data on distance travelled and vertical activity (rearing). Each mouse was placed in the top left corner facing the wall and allowed to roam freely in the arena for 3 min. Collected data were relayed to Versamax software (version 4.12) and presented coherently with Versadat program (version 3.02).

### 2.4 Hind-limb gait test

Locomotion was assessed in the mice by analysing hind-limb gait width and length using footprints [8]. Following two practice runs, the mouse's hind paws were dipped in black food dye and placed at the beginning of a paper-lined runway (50 cm long, 12 cm wide with 12 cm high) featuring a bright lamp at the start (adverse stimulus to induce movement) and a darkened ‘goal box’ (upturned, cloth-covered cage containing a mouse house) at the end. Two sets of prints were collected per mouse. Measurements were taken for 3–4 sets of footprints per run. Gait length was calculated from the distance between consecutive left and right footprints. Gait width was determined by measuring the distance between a left footprint and the perpendicular gait length of the two adjacent right footprints, and vice versa. The mean gait width and length of the two runs were calculated for each mouse.

### 2.5 Three-chamber socialisation test

Sociability was assessed in the mice using a previously described three-chamber socialisation test [24] with modifications. The social testing arena consisted of a white plastic rectangular box (55.5 cm × 36 cm × 33.5 cm) visually divided into three chambers (13.5 cm each) using a permanent marker. An unfamiliar, non-littermate, gender-matched wild-type (WT)^4^ mouse was placed in a small (8 cm diameter, 9.5 cm high) cylindrical wire cage (allowing contact but preventing fighting) in the left chamber of the arena for each test mouse. The test mouse was placed in the centre chamber and allowed to explore for 6 min. The number of entries into each chamber was recorded and quantified using a custom python script. Videos were captured using a camera (Logitech Webcam) mounted on a custom-made frame. Total chamber entries were divided by total test time (360 s) to give entries/s. Sociability index, described as time spent in the left chamber, was calculated by dividing left chamber entries by the total number of chamber entries achieved.

### 2.6 Nest building

Wellbeing measured via nest building was examined using a method designed from a previously reported assessment [17]. Mice did not undergo any practice prior to the test. Mice were individually caged overnight with cotton pillow nests (4.5 × 11.5 × 1.5, Pura PillowNest™) placed in the corner and no further environmental enrichment. The nest was photographed the following morning and quality of the nests was scored by a genotype-blinded researcher using the established rating scale as previously detailed [25].

### 2.7 Burrowing

Wellbeing measured through burrowing was assessed using a test designed from a previous report investigating a colorectal cancer mouse model [26]. Mice were individually housed in long cages (720 cm^2^) (containing paper bedding, mouse house and shredded paper) with ad libitum food and water for the duration of the test. Mice were acclimatized in darkness for the first hour and commenced burrowing during the second hour where the regular cage lid was replaced with the burrowing lid. Burrows consisted of plastic cups (Large (475 ml) Party Moments™ Plastic Tumblers, Adelaide, SA) filled with approximately 160 g of kitty litter (Chandler® Soft Natural Cat Litter, Adelaide, SA) and suspended at the opening from cage lids via zip ties (Ankom, 500 Pack Assorted Cable Ties (300 mm × 3.6 mm), Adelaide, SA). Burrow weights (g) were recorded before and after the test. Burrows with increased weight were excluded from analysis. Mice were acclimatized to the burrowing test apparatus and environment by allowing practice (in individual cages) the night before testing. Results were described as percentage of burrow contents removed within the hour.

### 2.8 Statistics

Statistical analysis was performed using GraphPad Prism V8.0.1 (GraphPad Software, La Jolla, CA). The statistical tests used to compare MPS IIIA mice with WT mice at each age comprised of one-way analysis of variance (ANOVA)^5^ with Bonferroni correction for multiple comparisons. *P* < 0.05 was considered statistically significant.

## 3 Results

### 3.1 Diminished memory and learning in MPS IIIA mice

**Fig. 1:**
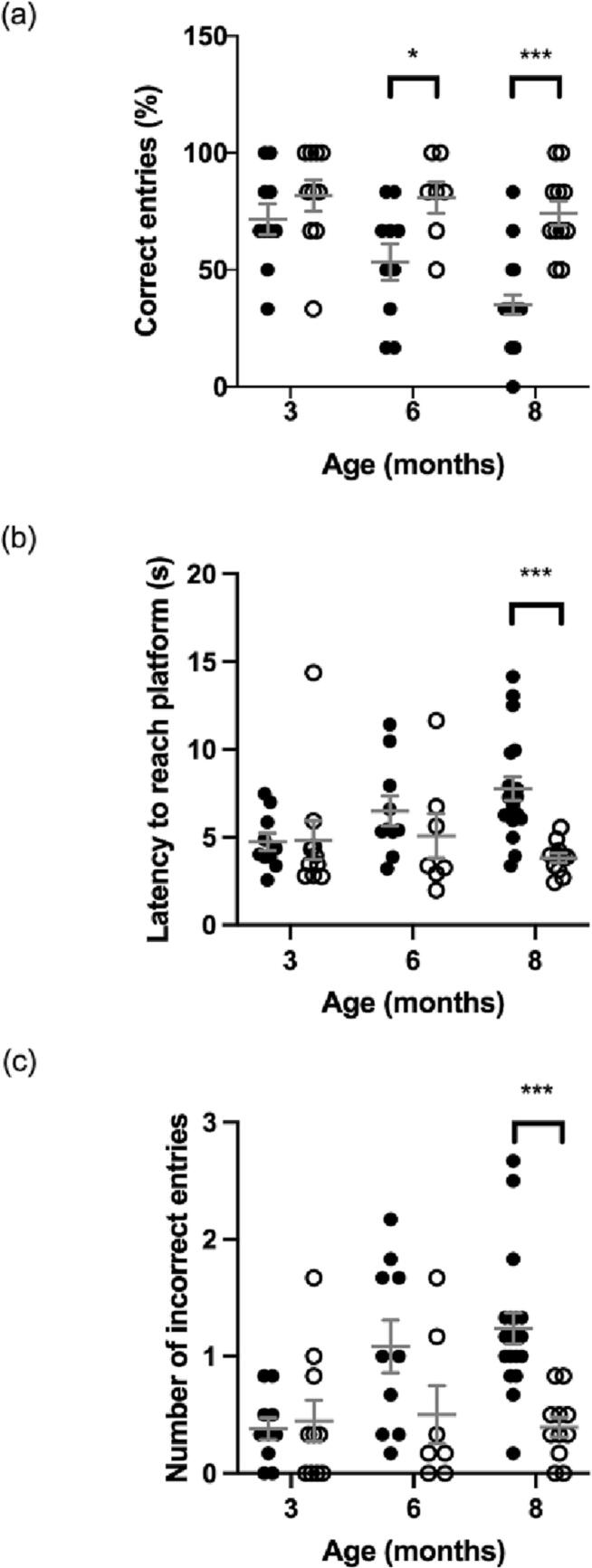
Memory and learning ability in the water cross-maze measured by correct entries (a) latency to reach platform (b) and incorrect entries (c) on the final day of assessment (day 5). MPS IIIA = black circles, WT = open circles. *n* = 10 WT and MPS IIIA at 3 months, *n* = 7 WT and 10 MPS IIIA at 6 months, *n* = 11 WT and 19 MPS IIIA at 8 months. *, *p* < 0.05, ***, *p* < 0.001. Individual data shown with mean ± SEM indicated by grey lines.

There is no significant difference in memory and learning between MPS IIIA and WT mice at 3 months of age. However, at 6 months of age MPS IIIA mice made significantly less correct entries (Fig. 1a) than their wildtype counterparts and by 8 months of age MPS IIIA mice displayed more incorrect entries, less correct entries and longer latency compared with WT mice (Fig. 1b,c).

### 3.2 Activity and locomotion progressively decreased in MPS IIIA mice

**Fig. 2:**
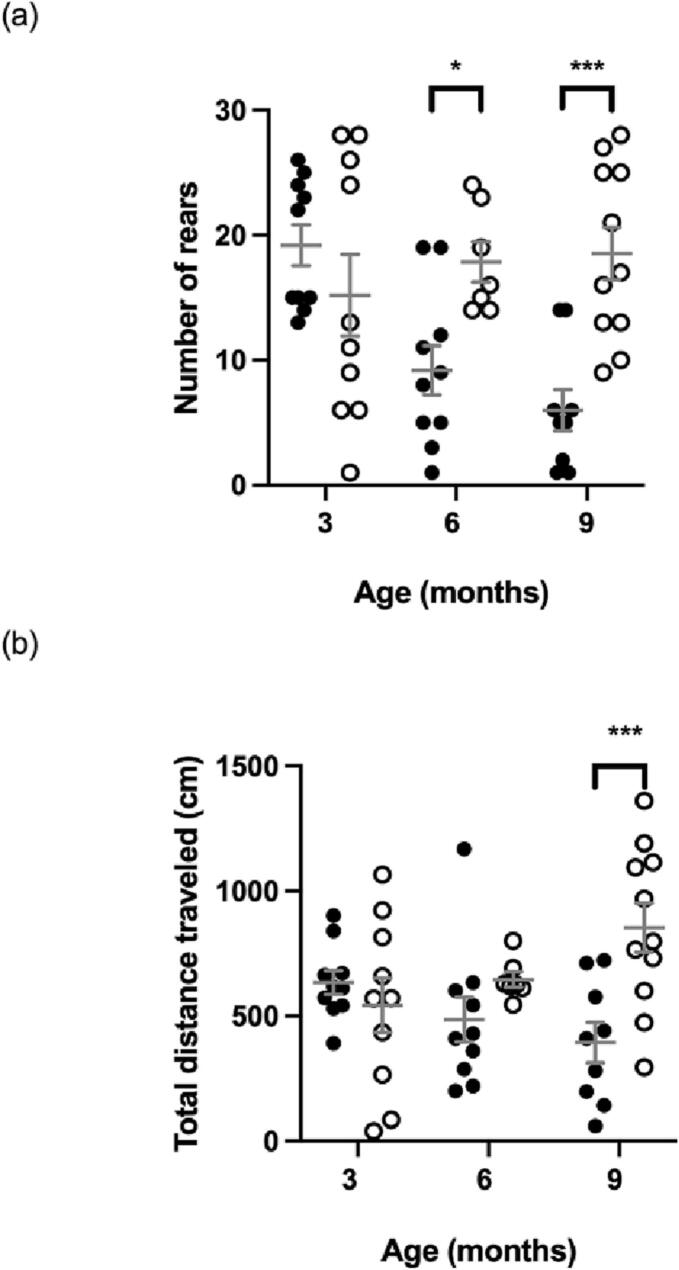
Activity in the open field measured as number of rears (a) and total distance travelled (b). MPS IIIA = black circles, WT = open circles. *n* = 10 WT and MPS IIIA at 3 months, *n* = 7 WT and 10 MPS IIIA at 6 months, *n* = 11 WT and 9 MPS IIIA at 9 months. *, *p* < 0.05, ***, *p* < 0.001. Individual data shown with mean ± SEM indicated by grey lines.

**Fig. 3:**
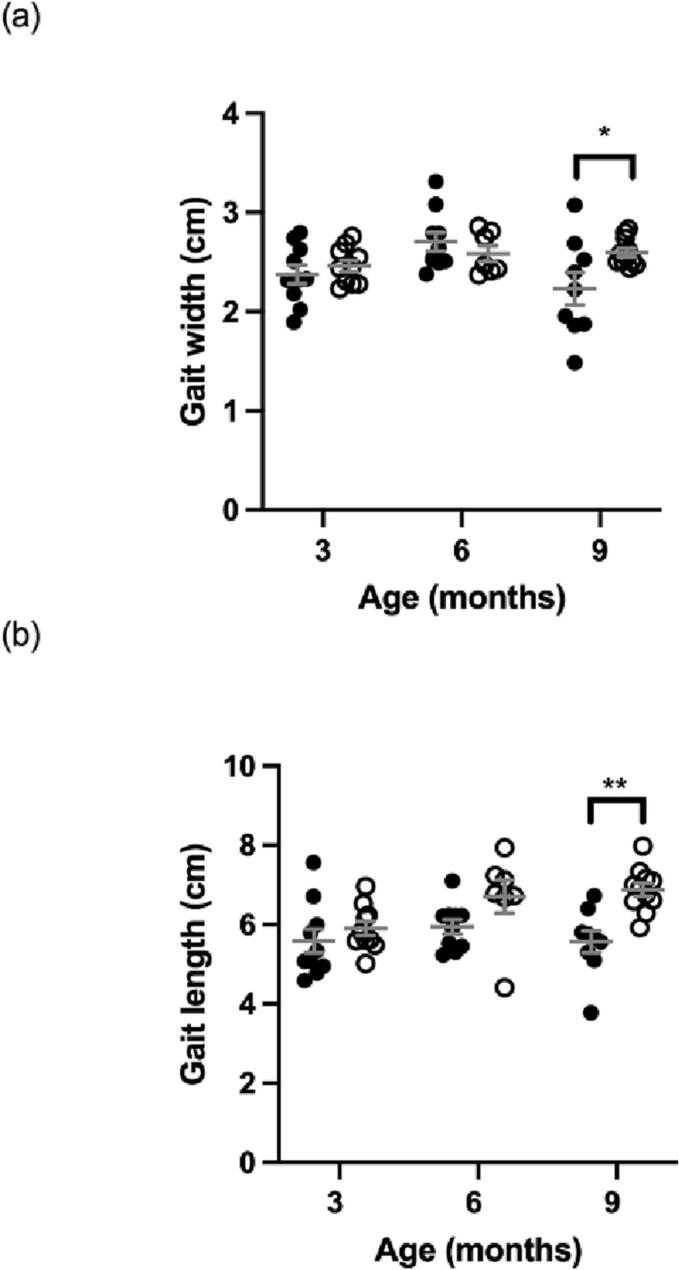
Locomotion in the gait assessment measured as average width (a) and length (b) of hind limb gait. MPS IIIA = black circles, WT = open circles. *n* = 10 WT and MPS IIIA at 3 months, *n* = 7 WT and 10 MPS IIIA at 6 months, *n* = 11 WT and 9 MPS IIIA at 9 months. *, p < 0.05, **, *p* < 0.01. Individual data shown with mean ± SEM indicated by grey lines.

Fig. 2, Fig. 3 show no significant differences between MPS IIIA and WT mice in activity and locomotion at 3 months of age, respectively. At 6 months, a significant difference is seen only for activity in MPS IIIA mice through decreased number of rears compared to age-matched WT mice. By 9 months, MPS IIIA mice displayed significant decreases in rears and distance travelled (Fig. 2) and a decrease in gait width and length is observed (Fig. 3).

### 3.3 Sociability unaffected in MPS IIIA mice

**Fig. 4:**
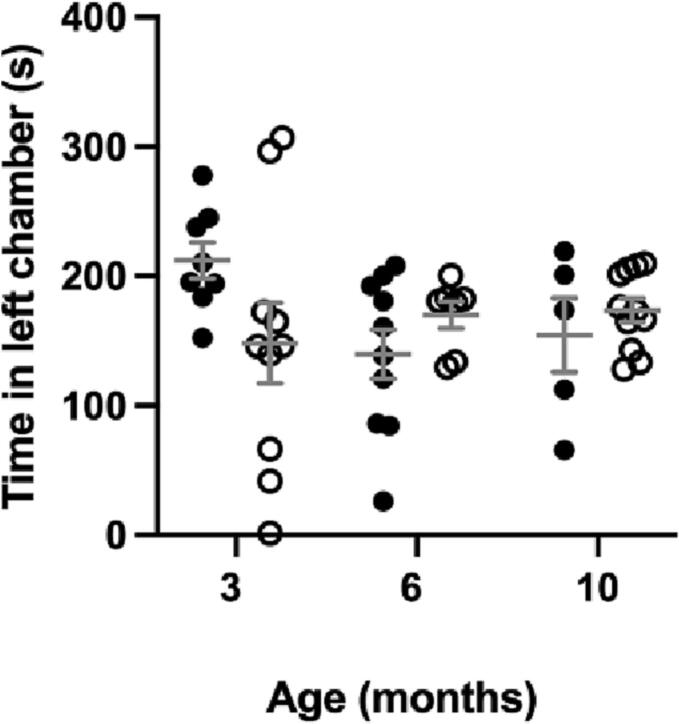
Sociability measured as time (seconds) spent in the left chamber containing a confined mouse unknown to the subject over 6 min. MPS IIIA = black circles, WT open circles. *n* = 10 WT and 8 MPS IIIA at 3 months, *n* = 7 WT and 10 MPS IIIA at 6 months, *n* = 11 WT and 5 MPS IIIA at 10 months. Individual data shown with mean ± SEM indicated by grey lines.

There is no significant difference in sociability between the MPS IIIA and WT mice at 3, 6 or 10 months of age (Fig. 4).

### 3.4 Wellbeing declined in older MPS IIIA mice

**Fig. 5:**
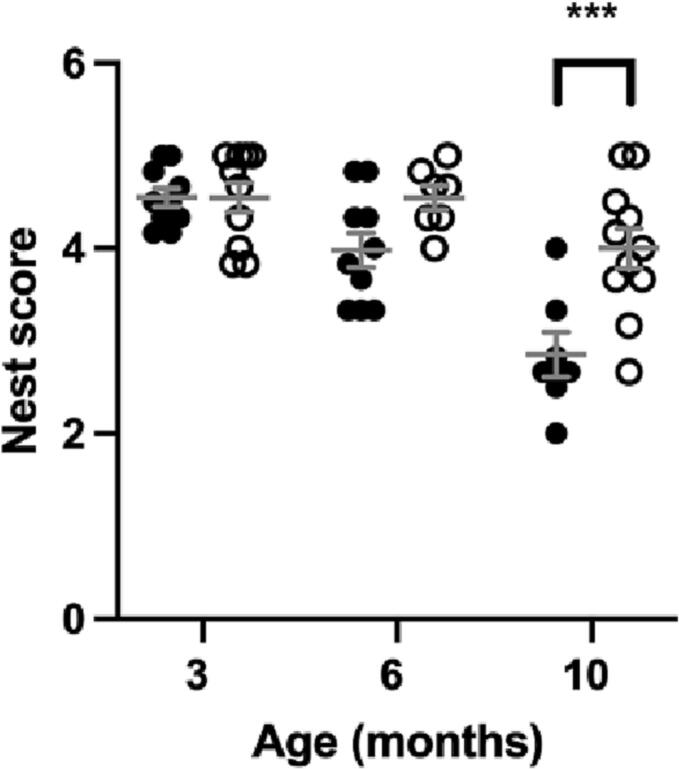
Nest building, measured as the average score obtained from a 5-point nest quality rating scale. MPS IIIA = black circles, WT = open circles. *n* = 10 WT and MPS IIIA at 3 months, *n* = 7 WT and 10 MPS IIIA at 6 months, *n* = 11 WT and 7 MPS IIIA at 10 months. ***, *p* < 0.001. Individual data shown with mean ± SEM indicated by grey lines.

**Fig. 6:**
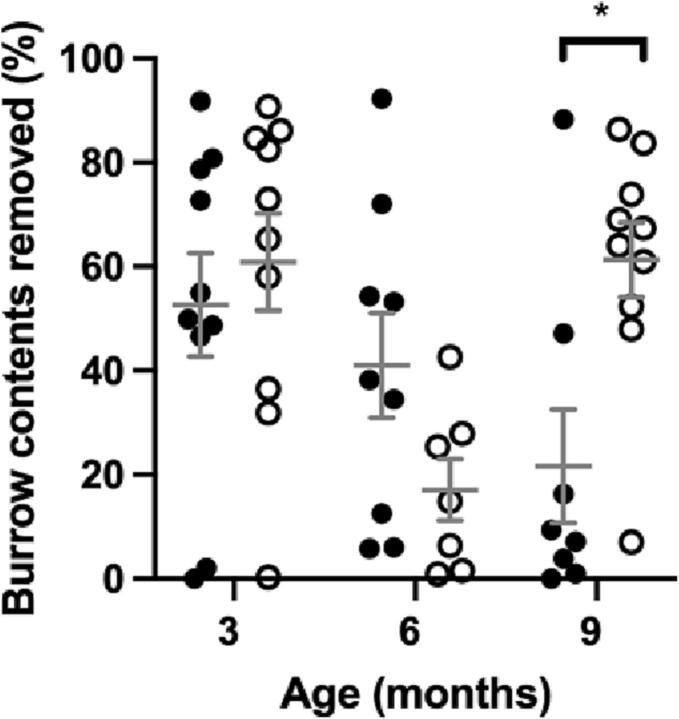
Burrowing measured as percentage of burrow contents removed in 1 h. MPS IIIA = black circles, WT = open circles. n = 10 WT and MPS IIIA at 3 months, n = 7 WT and 10 MPS IIIA at 6 months, n = 11 WT and 9 MPS IIIA at 9 months. *, *p* < 0.05. Individual data shown with mean ± SEM indicated by grey lines.

There is no significant difference in wellbeing between MPS IIIA and WT mice at 3 or 6 months of age as assessed through nest construction (Fig. 5) and burrowing (Fig. 6). MPS IIIA mice showed significantly decreased burrowing at 9 months and significantly poorer constructed nests at 10 months of age in comparison to age-matched WT mice.

## 4 Discussion

The water cross-maze revealed memory and learning impairment in MPS IIIA mice from mid-stage disease (6 months) (Fig. 1), concordant with previous studies [8,16] and reflecting human mid-stage disease cognitive skill regression and dementia [18]. To date, the water crossmaze has not been widely employed as the Morris Water Maze (MWM) has traditionally been used reporting memory and learning impairment in MPS IIIA mice as early as 4 months of age [12,27]. However, Fu et al. (2016) [7] showed 7.5 months was the earliest age of impairment. The discrepancy may be due to the MWM lacking the time-independent parameters of correct/incorrect entry employed in the water cross-maze, which are unaffected by non-cognitive variables such as swim-speed and provide additional measures of spatial learning [22]. Progressive retinal dystrophy has been reported in MPS IIIA mice from 3 months of age [28] and although we did not note corneal clouding in the MPS IIIA mice in agreement with earlier work [22], this may have an impact on performance of the MPS IIIA mice in the water cross-maze due to the dependence on visual cues.

Although not evaluated in this study, another approach to assess memory and learning is fear conditioning, where the animal freezes in response to an adverse stimulus, typically a shock (electrical) applied to the foot of the mouse. Contextual fear is measured with the mouse “freezing” in response, and this behavioural test has given consistent results in the MPS IIIA mouse model [9,15,29,30]. Fear conditioning has the distinct advantage of not relying on visual cues, but like the water cross maze it does require repetitive training components, and no measurable difference is seen in MPS IIIA mice until later in disease (8 months).

Impaired locomotion (gross motor coordination) at late-stage (9-months) (Fig. 3) concurs with Saville et al. (2021) [8] but disagrees with early-stage impairment reported in male mice [12,31]. This suggests male-specificity which may be masked in mixed-gender cohort testing. Locomotor impairment at late-stage in the mice is reflective of MPS IIIA patient gross motor deterioration whereas early-stage impairment is not reported in patients [3].

Quality of life is highlighted by parents and caretakers of MPS IIIA patients [21] as being important, but being largely dependent on physical, mental and social wellbeing are manifestations problematic to measure [32]. In rodents, wellbeing is assessed through performance of innate activities [33] such as nest building, which has been previously reported [17] to be normal in MPS IIIA mice tested up to 5 months of age. Burrowing, also an innate activity, has heretofore not been tested in MPS IIIA mice but has shown progressive decline in the dementing illness of prion disease [34,35]. Nest building and burrowing assessment results here show wellbeing is unaffected early in disease but deteriorates later as the disease progresses (Fig. 5, Fig. 6). Further research is needed to determine the utility of wellbeing assessments in the mice as they may be a useful surrogate measure of life quality in MPS IIIA.

Motivation, the driving force behind innate behaviours used to evaluate wellbeing, is reportedly linked to the striatal dopaminergic system (reviewed in [36]). Abnormalities in the striatal dopaminergic system caused by altered HS signalling have been reported in the MPS IIIA mouse [15], suggesting a possible cause for impaired wellbeing. The hippocampal component of the subcortex - dictating memory and learning - [37], and the cerebellum - controlling motor function (locomotion) [38] - both feature a significant HS storage burden in the MPS IIIA mouse from one month of age [10]. However, within the limitations of ages tested, functional deficit in the water cross-maze and hind-limb gait assessment are not observed until 6 and 9 months, respectively. This suggests the hippocampus and cerebellum can accommodate some HS before reaching a threshold at which time hippocampal and cerebellar function manifests as impaired memory and learning and gross motor coordination, respectively. Further analyses to explore the notion of HS thresholds in brain regions is needed to determine whether administering treatment before the threshold is reached provides additional neurocognitive benefit.

Hypoactivity, from mid-stage (6 months) (Fig. 2), concurred with Saville et al. (2021) [8], notably obtained from the same laboratory under similar testing conditions, but disagreed with hyperactivity reported in the MPS IIIA mouse by others [14,15]. Varying testing conditions between the studies are the likely cause of contradicting results, suggesting a lack of robustness for this assessment. Additionally, reliability of open field is uncertain given inconsistent reports of hyperactivity in MPS IIIA mice, despite it being a steady trait in MPS IIIA patients [3].

Unexpectedly, no social impairment at any stage of disease (Fig. 4) contrasted social deficits previously reported at 2 [15] and 7 months [13] using a three-chamber sociability-type assessment and at 4 months using reciprocal social interaction [17]. Like locomotion described above, this was in males, which could be impacted by their single-housing living arrangements or specifically unique to males due to their tendency towards aggression [17]. Thus, we suggest sociability is not a robust assessment for mix-gender studies and propose other autistic-like behaviours expressed in MPS IIIA such as hyperorality - the excessive mouthing of objects [19] - should be explored as an alternative. Hyperorality analysis may offer an autism-related behavioural analysis unaffected by gender-specific environmental and personality variables.

In conclusion, our data show that the water cross maze, hind-limb gait, nest building and burrowing are useful assessments to evaluate neurocognitive function in the MPS IIIA mouse model. They perform consistently and faithfully capture patient disease progression as reported in natural history studies, except for hind-limb gait assessment which is likely gender-biased. Open field and three-chamber sociability assessments are not robust and do not consistently reflect MPS IIIA pathology. Although we don't define the relationship between HS storage and behaviour, we postulate the idea of a substrate tolerance, defined as an amount of accumulated HS that the cell can tolerate before it manifests as neurocognitive impairment, which was only measurable later in disease.

## Funding

Kleopatra Pericleous is a recipient of a Faculty of Sciences Divisional Scholarship from the University of Adelaide.

## CRediT authorship contribution statement

**Kleopatra Pericleous:** Validation, Formal analysis, Investigation, Resources, Data curation, Writing – original draft, Visualization, Project administration. **Chantelle McIntyre:** Conceptualization, Methodology, Investigation. **Maria Fuller:** Conceptualization, Resources, Writing – review & editing, Supervision.

## Declaration of Competing Interest

None.

## Data Availability

Data will be made available on request.

## Footnotes

1 MPS IIIA, Mucopolysaccharidosis IIIA

2 SGSH, N-sulphoglucosamine sulphohydrolase

3 HS, heparan sulphate

4 WT, wild-type

5 ANOVA, analysis of variance

## References

[1] Neufeld E.F., Muenzer J. (2001).

[2] van de Kamp J.J., Niermeijer M.F., von Figura K., Giesberts M.A. (1981). Genetic heterogeneity and clinical variability in the Sanfilippo syndrome (types a, B, and C). Clin. Genet..

[3] Cleary M.A., Wraith J.E. (1993). Management of Mucopolysaccharidosis Type-III. Arch. Dis. Child..

[4] Escolar M.L., Jones S.A., Shapiro E.G., Horovitz D.D.G., Lampe C., Amartino H. (2017). Practical management of behavioral problems in mucopolysaccharidoses disorders. Mol. Genet. Metab..

[5] Delaney K.A., Rudser K.R., Yund B.D., Whitley C.B. (2014). Haslett, PA & Shapiro, EG 2014, methods of neurodevelopmental assessment in children with neurodegenerative disease: Sanfilippo syndrome. JIMD Reports..

[6] van der Lee J.H., Morton J., Adams H.R., Clarke L., Eisengart J.B., Escolar M.L., Giugliani R., Harmatz P., Hogan M., Kearney S., Muenzer J., Muschol N., Rust S., Saville B.R., Semrud-Clikeman M., Wang R., Shapiro E. (2020). Therapy development for the mucopolysaccharidoses: updated consensus recommendations for neuropsychological endpoints. Mol. Genet. Metab..

[7] Fu H., Cataldi M.P., Ware T.A., Zaraspe K., Meadows A.S., Murrey D.A., McCarty D.M. (2016). Functional correction of neurological and somatic disorders at later stages of disease in MPS IIIA mice by systemic scAAV9-hSGSH gene delivery. Mol. Therapy - Methods Clin. Develop..

[8] Saville J.T., Derrick-Roberts A.L.K., McIntyre C., Fuller M. (2021). Systemic scAAV9.U1a.hSGSH delivery corrects brain biochemistry in mucopolysaccharidosis type IIIA at early and later stages of disease. Hum. Gene Ther..

[9] Sorrentino N.C., Cacace V., De Risi M., Maffia V., Strollo S., Tedesco N., Nusco E., Romagnoli N., Ventrella D., Huang Y., Liu N., Kalled S.L., Choi V.W., De Leonibus E., Fraldi A. (2019). Enhancing the therapeutic potential of Sulfamidase for the treatment of Mucopolysaccharidosis IIIA. Mol. Therapy- Methods Clin. Develop..

[10] Saville J.T., Fuller M. (2020). Sphingolipid dyshomeostasis in the brain of the mouse model of mucopolysaccharidosis type IIIA. Mol. Genet. Metab..

[11] Gulinello M., Mitchell H.A., Chang Q., Timothy O’Brien W., Zhou Z., Abel T., Wang L., Corbin J.G., Veeraragavan S., Samaco R.C., Andrews N.A., Fagiolini M., Cole T.B., Burbacher T.M., Crawley J.N. (2019). Rigor and reproducibility in rodent behavioral research. Neurobiol. Learn. Mem..

[12] Soe K., Beard H., Neumann D., Trim P.J., Duplock S., Snel M.F., Hopwood J.J., Hemsley K.M. (2019). Early disease course is unaltered in mucopolysaccharidosis type IIIA (MPS IIIA) mice lacking α-synuclein. Neuropathol. Appl. Neurobiol..

[13] Gustavsson S., Sjöström E.O., Tjernberg A., Janson J., Westermark U., Andersson T., Makower A., Arnelöf E., Andersson G., Svartengren J., Ekholm C., Gelius S.S. (2019). Intravenous delivery of a chemically modified sulfamidase efficiently reduces heperan sulfate storage and brain pathology in mucopolysaccharidosis IIIA mice. Mol. Genetics Metabol. Reports..

[14] Langford-Smith A., Langford-Smith K.J., Jones S.A., Wynn R.F., Wraith J.E., Wilkinson F.L., Bigger B.W. (2011). Female mucopolysaccharidosis IIIA mice exhibit hyperactivity and a reduced sense of danger in the open field test. PLoS One.

[15] De Risi M., Tufano M., Alvino F.G., Alvino M.G., Torromino G., Gigante Y., Monfregola J., Marrocco E., Pulcrano S., Tunisi L., Lubrano C., Papy-Garcia D., Tuchman Y., Salleo A., Santoro F., Bellenchi G.C., Cristino L., Ballabio A., Fraldi A., De Leonibus E. (2021). Altered heparan sulfate metabolism during development triggers dopamine-dependent autistic-behaviours in models of lysosomal storage disorders. Nat. Commun..

[16] Kaidonis X., Byers S., Ranieri E., Sharp P., Fletcher J., Derrick-Roberts A. (2016). N-butyldeoxynojirimycin treatment restores the innate fear response and improves learning in mucopolysaccharidosis IIIA mice. Mol. Genet. Metab..

[17] Lau A.A., Tamang S.J., Hemsley K.M. (2018). MPS-IIIA mice acquire autistic behaviours with age. J. Inherit. Metab. Dis..

[18] Nidiffer F.D., Kelly T.E. (1983). Developmental and degenerative patterns associated with cognitive, behavioural and motor difficulties in the sanfilippo syndrome: an epidemiological study. J. Ment. Defic. Res..

[19] Rumsey R.K., Rudser K., Delaney K., Potegal M., Whitley C.B., Shapiro E. (2014). Acquired autistic behaviors in children with Mucopolysaccharidosis type IIIA. J. Pediatr..

[20] Wijburg F.A., Aiach K., Chakrapani A., Eisengart J.B., Giugliani R., Héron B., Muschol N., O’Neill C., Olivier S., Parker S. (2022). An observational, prospective, multicenter, natural history study of patients with mucopolysaccharidosis type IIIA. Mol. Genet. Metab..

[21] Porter K.A., O’Neill C., Drake E., Parker S., Escolar M.L., Montgomery S., Moon W., Worral C., Peay H.L. (2021). Parent experiences of sanfilippo syndrome impact and unmet treatment needs: a qualitative assessment. Neurol. Therapy..

[22] Roberts A.L., Rees M.H., Klebe S., Fletcher J.M., Byers S. (2007). Improvement in behaviour after substrate deprivation therapy with rhodamine B in a mouse model of MPS IIIA. Mol. Genet. Metab..

[23] Saville J.T., Lehmann R.J., Derrick-Roberts A.L.K., Fuller M. (2016). Selective normalisation of regional brain bis(monoacylglycero)phosphate in the mucopolysaccharidosis 1 (Hurler) mouse. Exp. Neurol..

[24] El-Kordi A., Winkler D., Hammerschmidt K., Kastner A., Krueger D., Ronnenberg A., Ritter C., Jatho J., Radyushkin K., Bourgeron T., Fischer J., Brose N., Ehrenreich H. (2013). Development of an autism severity score for mice using Nlgn4 null mutants as a construct-valid model of heritable monogenic autism. Behav. Brain Res..

[25] Deacon R.M.J. (2006). Assessing nest building in mice. Nat. Protoc..

[26] Chartier L.C., Howarth G.S., Lawrance I.C., Trinder D., Barker S.J., Mashtoub S. (2017). Emu oil improves clinical indicators of disease in a mouse model of colitis-associated colorectal cancer. Dig. Dis. Sci..

[27] Crawley A.C., Gliddon L.B., Auclair D., Brodie S.L., Hirte C., King B.M., Fuller M., Hemsley K.M., Hopwood J.J. (2006). Characterization of a C57BL/6 cogenic mouse strain of mucopolysaccharidosis type IIIA. Brain Res..

[28] Intartaglia D., Giamundo G., Marrocco E., Maffia V., Salierno F.G., Nusco E., Fraldi A., Conte I., Sorrentino N.C. (2020). Retianl degeneration in MPS-IIIA mouse model. Front. Cell Develop. Biol..

[29] Capuozzo A., Montefusco S., Cacace V., Sofia M., Esposito A., Napolitano G., Nusco E., Polishchuk E., Pizzo M.T., De Risi M., De Leonibus E., Sorrentino N.C., Medina D.L. (2022). Fluoxetine ameliorates mucopolysaccharidosis type IIIA. Mol. Ther..

[30] Monaco A., Maffia V., Sorrentino N.C., Sambri I., Ezhova Y., Giuliano T., Cacace V., Nusco E., De Risi M., De Leonibus E., Schrader T., Klärner F., Bitan G., Fraldi A. (2020). The amyloid inhibitor CLR01 relieves autophagy and ameliorates neuropathology in a severe lysosomal storage disorder. Mol. Ther..

[31] Fraldi A., Hemsley K., Crawley A., Lombardi A., Lau A., Sutherland L., Auricchio A., Ballabio A., Hopwood J.J. (2007). Functional correction of CNS lesions in an MPS-IIIA mouse model by intracerebral AAV-mediated delivery of sulfamidase and SUMF1 genes. Hum. Mol. Genet..

[32] Eiser C., Morse R. (2001). A review of measures of quality of life for children with chronic illness. Arch. Dis. Child..

[33] Jirkof P. (2014). Burrowing and nest building behavior as indicators of well-being in mice. J. Neurosci. Methods.

[34] Mallucci G.R., White M.D., Farmer M., Dickinson A., Khatun H., Powell A.D., Brandner S., Jefferys J.G., Collinge J. (2007). Targeting cellular prion protein reverses early cognitive deficits and neurophysiological dysfunction in prion-infected mice. Neuron..

[35] Cunningham C., Campion S., Lunnon K., Murray C.L., Woods J.F.C., Deacon R.M.J., Rawlins J.N.P., Perry V.H. (2009). Systemic inflammation induces acute behavioral and cognitive changes and accelerates neurodegenerative disease. Biol. Psychiatry.

[36] Salamone J.D., Correa M. (2012). The mysterious motivation functions of mesolimbic dopamine. Neuron..

[37] Jarrard L.E. (1993). On the role of hippocampus in learning and memory in the rat. Behav. Neural Biol..

[38] Gowen E., Miall R.C. (2007). The cerebellum and motor dysfunction in neuropsychiatric disorders. Cerebellum.

